# Case Report: A Rare Case of Pembrolizumab-Induced Bullous Pemphigoid

**DOI:** 10.3389/fimmu.2021.731774

**Published:** 2021-09-14

**Authors:** Xiaoyan Zhang, Dongjiang Sui, Dong Wang, Lina Zhang, Ruiyan Wang

**Affiliations:** ^1^Department of Pulmonary and Critical Care Medicine, Air Force Medical Center, Beijing, China; ^2^Department of Dermatology, Air Force Medical Center, Beijing, China

**Keywords:** bullous pemphigoid, pembrolizumab, programmed cell death protein 1, immunotherapy, glucocorticoid

## Abstract

The programmed cell death protein 1 inhibitor pembrolizumab, an immune checkpoint inhibitor, has subsequently been approved for the treatment of a wide variety of malignant tumors. Compared with conventional chemotherapy, immunotherapy is associated with a unique set of immune reactions, known collectively as immune-related adverse events. Although often mild, dermatologic toxicity can occasionally be high grade and potentially life-threatening. Here we describe a rare case of bullous pemphigoid (BP) associated with pembrolizumab. A 79-year-old male patient presented with scattered erythema, papules, blisters, and pruritus after pembrolizumab treatment. Then, the rash gradually aggravated and spread to the whole body. The extensive edematous erythema, blisters, bullae, and blood blisters were loose and easy to rupture, forming an erosive surface and with pruritus and obvious pain. The hemidesmosomal protein BP180 (type XVII collagen) was detectable in the serum, and the histological examination diagnosis was bullous pemphigoid. After 10 days of glucocorticoid (methylprednisolone, iv, 80 mg/day) treatment, new blister formation ceased. We need to increase the awareness on and facilitate the earlier identification of the cutaneous adverse effects of BP with immunotherapy so that treat can begin early in order to limit the duration and severity of toxicity.

## Introduction

Pembrolizumab is a humanized monoclonal antibody IgG4 programmed cell death protein 1 (PD-1) antagonist. As a new drug of immune checkpoint inhibitor, pembrolizumab has shown great promising prospects in clinical antitumor immunotherapy and has rapidly become the first-line therapy for a variety of advanced malignancies. It has been recommended in antitumor guidelines of many countries and opened a new era of tumor immunotherapy (1). While immune checkpoint inhibitors can enhance the immune system and cause cells to attack tumor cells, they can also cause cells to attack normal cells in the body, causing a series of inflammatory diseases mimicking autoimmune diseases, some of which may be serious. These major adverse effects are termed “immune-related adverse events” (irAEs) and present with organ-specific tissue inflammation (2–4). Here we report a case of bullous pemphigoid (BP) associated with pembrolizumab, emerging with a potentially serious dermatologic toxicity, and we will use this case to highlight the diagnosis and management of cutaneous irAEs associated with checkpoint inhibitors.

## Case Report

**Figure 1:**
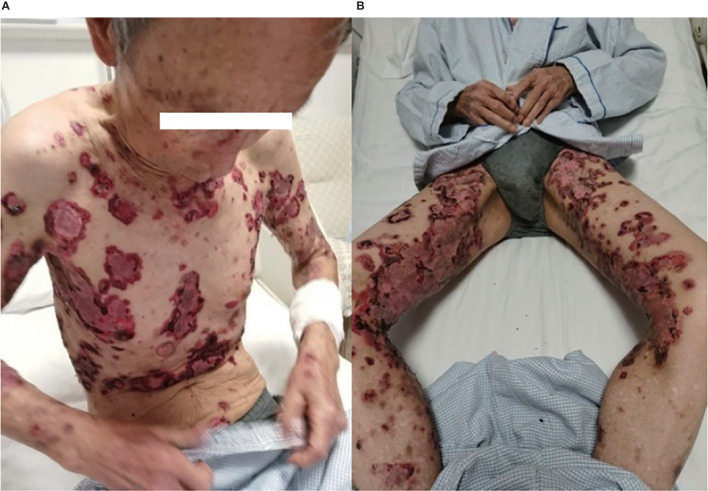
**(A, B)** Skin lesions on admission.

In May 2019, a 79-year-old male patient underwent resection of the left kidney and ureter for invasive high-grade papillary urothelial carcinoma. The tumor size was 1.2 × 1 × 0.2 cm, and the tumor TNM scale was T1N0M1. In January 2020, multiple round nodules of different sizes can be seen in both lungs by CT and PET-CT, with a smooth boundary. The largest one is located in the left lower lung, about 2.2 × 1.8 cm. From February to September 2020, he had received 10 cycles of pembrolizumab therapy (100 mg, iv, q21d) because of lung metastatic carcinoma. From April 2020, erythema and pimples accompanied by pruritus appeared successively on the trunks and limbs of the patient. After symptomatic treatment, the rash subsided and appeared repeatedly. From November 2020, the rash aggravated gradually; macroscopically, the lesions appeared as strained blisters filled with serous fluid and blood on erythematous skin, spreading throughout the body. The blood bullae were big, loose, and easy to break, forming the erosive surface (**Figures 1A, B**), and accompanied by itchy and obvious pain. According to the descriptions of select cutaneous immune-related adverse events as defined by the CTCAE, version 5.0 (Common Terminology Criteria for Adverse Events), given the blisters of the patient covering >30% of body surface area which limit his self-care/activities of daily living, his disease grade was classified as grade 3.

**Figure 2:**
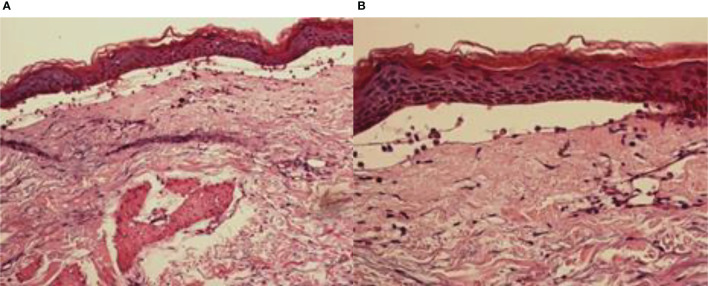
Skin pathology: **(A)** H&E, ×200; **(B)** H&E, ×400.

**Figure 3:**
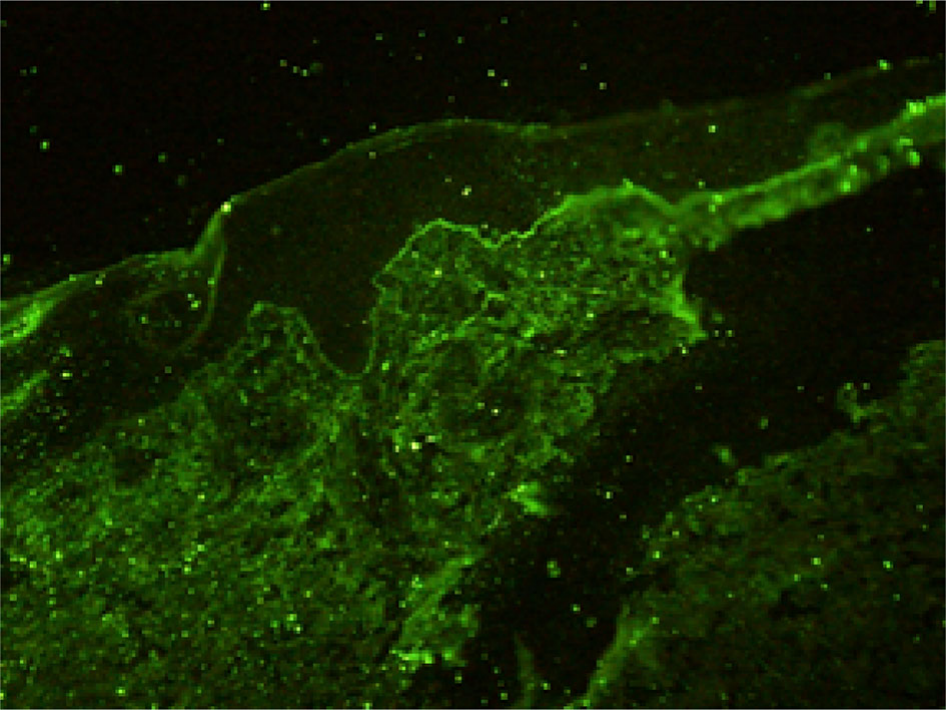
IgG immunoglobulin deposits along the dermo-epidermal junction.

**Figure 4:**
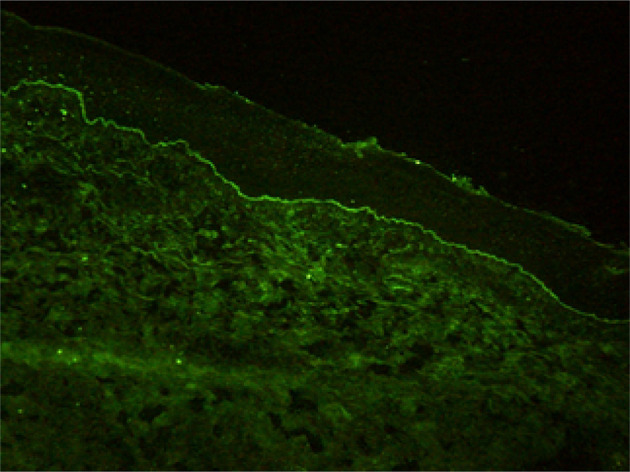
C3 deposits along the dermo-epidermal junction.

Histopathological findings showed epidermal hyperplasia and edema, subepidermal blister formation, numerous eosinophils in the blister, and obvious eosinophil infiltration around the superficial dermis vessels (**Figures 2A, B**). Immunofluorescence was positive for IgG (**Figure 3**) and C3 (**Figure 4**). The hemidesmosomal protein BP180 (type XVII collagen) was detectable in the serum, with 137.32 U/ml titer by enzyme-linked immunosorbent assay (ELISA) method. BP230 detection was normal. Taken together, the clinical presentation and laboratory findings were consistent with the diagnosis of BP. According to Naranjo’s adverse drug reaction assessment with a score of 8 (**Table 1**), BP probably caused by pembrolizumab was considered.

**Table 1:** Naranjo’s assessment scale in the evaluation of adverse drug reaction (ADR).

Related issues	Results	Score
1. Is there any previous conclusive report on this ADR?	No	0
2. Does the ADR occur after the use of suspicious drugs?	Yes	2
3. Does the ADR get remission after drug withdrawal or anti-drug application?	Yes	1
4. Is the ADR repeated after the use of the suspected drug again?	Yes	1
5. Is there any other reason that can cause the ADR alone?	No	2
6. Does the ADR recur after placebo?	Unknown	0
7. Does the drug reach a toxic concentration in the blood or other body fluids?	Unknown	0
8. Does the ADR aggravate with the increase of dose or alleviate with the decrease of dose?	Yes	1
9. Has the patient ever been exposed to the same or similar drugs and had similar reactions?	No	0
10. Is there any objective evidence to confirm the reaction?	No	1
Total score		*r*

Naranjo’s score: ≥ 9 points, definite; 5 – 8 points, probable, 1 – 4 points, possible; ≤ 0 points, doubtful.

Our differential diagnosis included paraneoplastic pemphigus (PNP). Direct immunofluorescence did not show a deposition of immunoglobulins at the cell surface of keratinocytes. By employing PET-CT, multiple hypermetabolic metastases in the lungs were detected on January 10, 2020, while the PET-CT image taken on November 19, 2020 showed that the original lesions were not clearly displayed. This suggested that immunotherapy was effective in the early stage, and the tumor achieved clinical complete remission (CR) after the treatment. No typical malignant hypermetabolic lesions were found in the rest of the body, so PNP can be excluded. BP can be caused by some drugs, including furosemide and anti-DPP4 inhibitors. This patient was not prescribed with these drugs.

**Figure 5:**
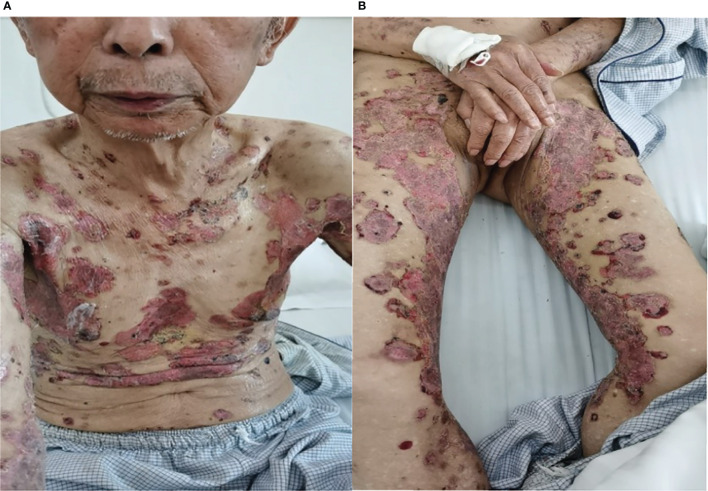
**(A, B)** Skin lesions on the 8th day after glucocorticoid treatment.

After 3 days of glucocorticoid treatment (methylprednisolone, iv, 80 mg/day, 1.25 mg/kg/day), the cutaneous lesions improved. In some parts, the blister fluid was absorbed and the skin surface shrunk, the erosive surface began to scab and heal, and the color of erythema became pale. After 10 days, all the blisters disappeared, and new bullae formation ceased. All the superficial erosions were scabby and most of the scabs fell off, leaving dark red pigmentation spots (**Figures 5A, B**). After 2 weeks of glucocorticoid treatment, the methylprednisolone dose was changed to oral administration and gradually tapered. At 1 month later, BP180 detection was normal with 8.49 U/ml titer.

**Figure 6:**
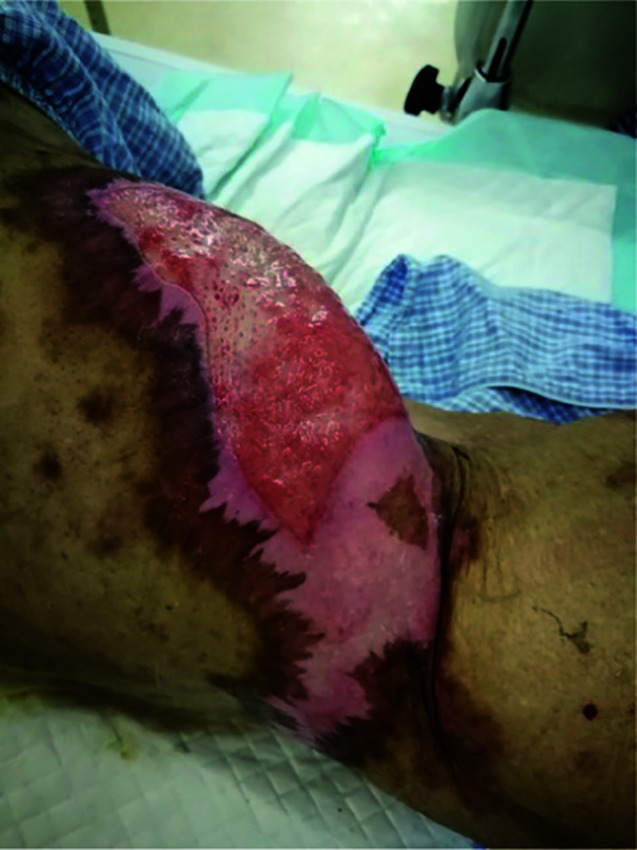
Bullous pemphigoid reappearance after steroid tapering and continuous therapy for 5 months; skin of left hip joint.

In February 2021, when methylprednisolone has been used as treatment for 4 months and was reduced to 24 mg/day, several blood blisters fused together with a size of about 20 × 7cm, which appeared in the skin of the left hip joint of the patient. BP180 was redetected, suggesting BP recurrence. The dose of methylprednisolone was increased to 44 mg for treatment. The blood blisters were broken and formed a big ulcer with yellow green pyoderma, and regular debridement and treatment with topical ointment, such as human epidermal growth factor gel, halometasone cream, and silver ion dressing, were continued for several months. So far (in July 2021), the skin lesions were not healed (**Figure 6**), the dose of methylprednisolone was reduced to 25 mg/day, and the monitored BP180 was in the normal range. In March 2021, the patient began to have a cough and low fever, and the lung CT showed invasive pulmonary fungal infection; he received antifungal treatment. During the antifungal treatment, for three times the lung CT showed that there was no lung carcinoma, and the carcinoma was still in CR.

## Discussion

As a new anti-tumor drug, pembrolizumab is an IgG4 antibody which antagonizes the PD-1 receptor. It has been approved for the treatment of a wide variety of malignant tumors, including melanoma, non-small cell lung cancer, head and neck squamous cell carcinoma, classical Hodgkin’s lymphoma, urothelial carcinoma, gastric cancer, and cervical cancer. However, immune checkpoint inhibitors may abnormally enhance the normal immune response and cause T cells to attack the tumor cells in the body, causing a series of toxic reactions similar to autoimmune diseases (5, 6). Although the immune-related toxicity of pembrolizumab events are rare, serious adverse events, such as BP, can occur if it involves multiple systems and has a wide range of influence. It has been reported that the rash caused by the PD-1 inhibitor is often seen as pruritus or atypical macular papules in the early stage, and mucosal involvement is rare (7–9). The incidence of high-grade cutaneous toxicity with PD-1 inhibitors is low (approximately 1–2%) (10). Direct immunofluorescence showed the linear deposition of IgG and C3 at the dermo-epidermal junction. ELISA is the most commonly used antibody to detect the dermal component BP 180 (11), and in our case, BP 180 antibody was detected. The immune checkpoint inhibitors may determine the depletion of regulatory T cells (TREGs) that, in turn, leads to the proliferation of antigen-specific B cells and can facilitate the humoral response at the germinal center lever of lymphatic follicles. All these caused the production of the BP180 antibody (12). Sometimes the antibody to BP230 can be detected. Although this is the same pathomechanism believed to cause conventional BP, it is still unclear how anti-PD-1/PD-L1 immunotherapy facilitates this reaction (9).

Differently from other BP induced by traditional drugs, BP related to anti- PD-1/PDL1 may continue for several months after drug withdrawal. The incubation period of BP induced by PD-1 inhibitors is longer. Bullous lesions usually appear within the first 20–32 weeks of treatment; the mean time is 39 weeks, with some cases taking even more than 80 weeks. This may be related to the sustained immune activation (12, 13). BP is of high-grade dermatologic toxicity and potentially life-threatening if not be treated early. The delayed effect of the PD-1 inhibitor induced BP and may be the lasting effect of the drug itself, or the pathomechanism of the PD-1 inhibitor induced BP, which involves the key node in the pathogenesis of BP. Once triggered, it will form a positive feedback cycle and can no longer stop by itself. In our case, the onset of BP induced by PD-1 inhibitors is about 28 weeks.

Pembrolizumab has changed the treatment of malignant tumor, but more and more patients have immune-related adverse events. BP is of high-grade cutaneous toxicity to patients and potentially life-threatening. Although the continued use of anti-PD-1 drugs may aggravate BP, discontinuation does not lead to complete remission. These patients may still need intermittent or continuous treatment of BP. According to the severity of BP, glucocorticoid can be used locally or systematically. The systemic application of glucocorticoids can improve the prognosis of pemphigoid and reduce the mortality to 25–45%. After combining with other adjuvant therapy, the mortality was less than 10%. The cause of death of pemphigoid is very complex. Most scholars believed that respiratory tract infection and hormone adverse effects are the main cause. Some people think that the prognosis of pemphigoid is related to systemic failure and coronary heart disease. A few patients died of complications of long-term systemic use of high-dose corticosteroids and immunosuppressants, and infection was the most common cause of death (3).

## Conclusion

With the use of pembrolizumab, clinicians should have increased awareness and facilitate the earlier identification of this rare but potentially serious cutaneous irAEs. Because cutaneous irAEs may occur late and even occur after pembrolizumab therapy has been completed, clinicians should keep in mind and evaluate carefully the new cutaneous adverse effects. Early treatment of BP is important to limit the duration and severity of toxicity. It is important to prevent and/or reduce interruptions in potentially life-saving cancer therapy.

## Data Availability Statement

The original contributions presented in the study are included in the article/supplementary material. Further inquiries can be directed to the corresponding author.

## Ethics Statement

Written informed consent was obtained from the individual(s) for the publication of any potentially identifiable images or data included in this article.

## Author Contributions

XZ and DS contributed to data collection, data analysis, data interpretation, and manuscript preparation. DW and LZ contributed to data analysis and data interpretation. RW contributed to data collection. XZ contributed to data review and interpretation. All authors contributed to the article and approved the submitted version.

## Conflict of Interest

The authors declare that the research was conducted in the absence of any commercial or financial relationships that could be construed as a potential conflict of interest.

## Publisher’s Note

All claims expressed in this article are solely those of the authors and do not necessarily represent those of their affiliated organizations, or those of the publisher, the editors and the reviewers. Any product that may be evaluated in this article, or claim that may be made by its manufacturer, is not guaranteed or endorsed by the publisher.
